# THE DYNAMICS OF ECONOMIC INSECURITY THROUGHOUT LATER LIFE: DISPARITIES BY GENDER AND RACE/ETHNICITY

**DOI:** 10.1093/geroni/igae098.0464

**Published:** 2024-12-31

**Authors:** Seokmin Kim, Sung Park, Jan Mutchler

**Affiliations:** University of Massachusetts Boston, Boston, Massachusetts, United States; University of Massachusetts Boston, Boston, Massachusetts, United States; University of Massachusetts Boston, Boston, Massachusetts, United States

## Abstract

Older Americans face significant economic hardships, and their risk of poverty increases with age. But it remains unclear whether the 65+ population, which includes a sizable number with incomes too high to qualify for means-tested public assistance programs, has the resources to meet an adequate standard of living. Using 2010-2020 data from the Health and Retirement Study linked to the corresponding years of the Elder Index (EI), this project investigates heterogeneity in economic insecurity among older adults aged 65+ as they age, by gender and race/ethnicity. We examine transitions in living above/below the EI and time spent living below the EI. We also study the dynamic relationship between respondents’ employment activity and their trajectories of economic insecurity. Results suggest that at least half of respondents live below the EI at some point in later life, and a quarter of them experience sustained economic insecurity throughout the entire observation period. Individuals in these economically insecure categories are more likely to be female, non-White, and have limited or no work experience. These disparities grow over time as individuals approach their 80s, driven partly by the intensity of work experience prior to age 60, despite increasing employment activity in later life. The findings illustrate the growing disadvantage in economic well-being for older females and racial/ethnic minorities, and the influence of earlier life economic activity on financial insecurity and instability in later life.

